# Global ozone depletion and increase of UV radiation caused by pre-industrial tropical volcanic eruptions

**DOI:** 10.1038/s41598-019-45630-0

**Published:** 2019-07-01

**Authors:** Hans Brenna, Steffen Kutterolf, Kirstin Krüger

**Affiliations:** 1 Section for Meteorology and Oceanography, Department of Geosciences, University of Oslo, P.O. Box 1022 Blindern, 0315 Oslo, Norway; 2 GEOMAR | Helmholtz Centre for Ocean Research Kiel, Wischhofstrasse 1-3, 24148 Kiel, Germany

**Keywords:** Palaeoclimate, Volcanology, Atmospheric dynamics, Atmospheric chemistry

## Abstract

Large explosive tropical volcanic eruptions inject high amounts of gases into the stratosphere, where they disperse globally through the large-scale meridional circulation. There is now increasing observational evidence that volcanic halogens can reach the upper troposphere and lower stratosphere. Here, we present the first study that combines measurement-based data of sulfur, chlorine and bromine releases from tropical volcanic eruptions with complex coupled chemistry climate model simulations taking radiative-dynamical-chemical feedbacks into account. Halogen model input parameters represent a size-time-region-wide average for the Central American eruptions over the last 200 ka ensuring a comprehensive perspective. The simulations reveal global, long-lasting impact on the ozone layer affecting atmospheric composition and circulation for a decade. Column ozone drops below 220 DU (ozone hole conditions) in the tropics, Arctic and Antarctica, increasing biologically active UV by 80 to 400%. Our model results could potentially be validated using high-resolution proxies from ice cores and pollen records.

## Introduction

### Halogens and Sulfur from Volcanic Eruptions

Large amounts of material are ejected into the atmosphere by large, explosive volcanic eruptions. This material consists of tephra (explosively fragmented magma), as well as volatiles (gases) in variable concentrations. Significant volcanic volatile compounds, influencing atmospheric conditions, are carbon dioxide, sulfur dioxide, which form sulfuric acid aerosols over a period of about one month, and halogens (e.g. chlorine and bromine), that play a major role in ozone chemistry^1,2^. Halogen compounds that reach the stratosphere contribute to catalytic ozone loss by releasing halogen radicals and interacting with enhanced sulfuric acid aerosol leading to increased ozone loss through heterogeneous chemistry^3^.

**Figure 1:**
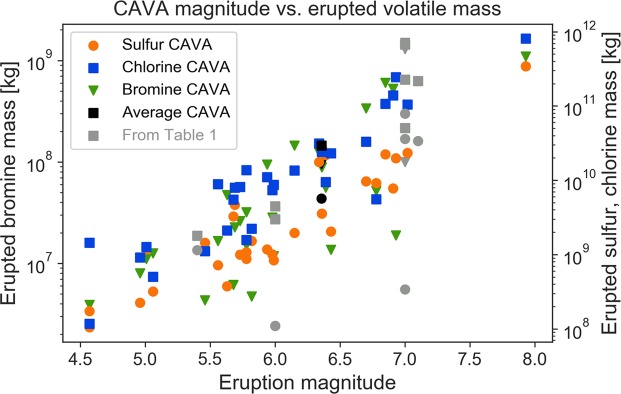
Erupted mass of sulfur, chlorine and bromine as a function of eruption magnitude for the full CAVA data set^14,15^. Black symbols indicate the average CAVA values and gray symbols represent the eruptions listed in Table 1.

Tropical eruptions with eruptive columns rapidly penetrating the tropopause directly inject volatiles in the form of gas, ice or salts precipitated on ash surfaces into the stratosphere where they are distributed globally by the large-scale meridional overturning circulation. Sulfuric acid aerosols and halogens then impact radiation, chemistry and dynamics of the stratosphere. The effects of volcanic sulfur emissions on the global climate is widely studied and accepted^4,5^. Although progress has been made on modeling volcanic chemistry interactions over the last years^6–11^, the complex impacts and feedbacks of volcanic halogens on radiation, chemistry, and dynamics are still under-explored. Total erupted sulfur and halogen masses can range from tons to megatons (1 million metric tons, Mt)^12–16^ (Fig. 1, Table 1). While most erupted sulfur is injected to the stratosphere, halogens are easily scavenged by water and ice in the rapidly ascending eruption column, leaving a large uncertainty concerning how much of the emitted halogen is injected into and remain in the stratosphere^17^.

**Table 1:** Comparison of the volatile release from the average CAVA eruption with the largest eruptions of the satellite era and some large eruptions of the Holocene.

Eruption	Petrologic SO_2_* (Mt)	Stratospheric SO_2_^*^ (Mt)	Petrologic Cl (Mt)	Stratospheric Cl (Mt)	Petrologic Br (Mt)	Estimated Br (Mt)^**^	Total erupted mass (Gt)	Magnitude (M_v_)^51^
Average CAVA	11.4^14^	—	29.25^15^	—	0.095^15^	—	22.8^15^	6.4
Pinatubo (1991)	0.22^21^	17 (±3)^22^	3^22^–4.5^21^	Negligible^26^	—	—	10^21^	6.0
El Chichón (1982)	2.31^93^	15 (±30%)^94^	1.8^20^	0.04^24^	—	—	2.3^95^	5.4
Santorini (~1600 BCE)	0.68–72^10^	—	50.6–675^10^	—	—	0.1–1.5^10^	90^10^	7.0
Samalas (1257)	158^47^	—	227^47^	—	—	1.3^47^	100^47^	7.0
Mt Mazama (~5600 BCE)	68^46^	58–146^45^	217^46^	8.1^45^	—	—	126^46^	7.1

*Equivalent SO_2_ masses are calculated from the corresponding mass of atomic sulfur where necessary.

**Estimated bromine is based on experimentally determined ratios (e.g. Cl/Br) and measured Cl values, assuming that the ratios are constant for all magmatic systems.

The hypothesis that volcanic chlorine could impact the ozone layer originated in the 1970s^18^. An early investigation was carried out using simplified estimates of chlorine injection and dispersion^19^. These calculations indicated that chlorine from major volcanic eruptions could deplete ozone by several percent of the total column, such as the 1883 Krakatoa eruption with about 7% and the 1963 Agung eruption by just 1% column ozone reduction.

The two largest eruptions in the satellite era, El Chichón and Pinatubo, released approximately1.8^20^ and 3–4.5^21–23^ Mt of chlorine respectively (determined through petrological estimates) and provide two well-observed test cases of volcanic halogen surveillance. After the 1982 El Chichón eruption evidence from aircraft measurements indicated that the volcanic explosion injected at least 0.04 Mt of HCl (equal to 9% of the global stratospheric chlorine burden at that time) into the stratosphere^24^. In addition, NaCl (halite) particles, which release HCl through reactions with sulfuric acid aerosol, were detected in the stratospheric volcanic cloud^25^. In contrast, after the 1991 Pinatubo eruption, the measured increase of HCl in the plume was substantially less than that following El Chichón 1982^26^. Pinatubo was special in the sense that the eruption happened at the same time and location as a typhoon hitting the Philippines, possibly leading to effective wash out of halogens^1,12^. Nonetheless, the increased heterogeneous chemistry caused by the Pinatubo aerosol cloud depleted global ozone, peaking at a 7 DU (Dobson Units, a measure of the thickness of the ozone column)^27^ a ~2.5% reduction of global ozone. The difference in volatile injection from El Chichón and Pinatubo points to a strong dependence on the characteristic and size of the specific volcanic eruption including the composition of the magma^15,28^ and surrounding environmental conditions^12,26,29^ as well as spatial and temporal variations (i.e., latitude, season, and injection height)^30–32^. Furthermore, the volcanic impact on ozone depends on the background halogen content of the atmosphere^12,33^ with implications for the impact on radiation and climate.

A number of observations of halogen injections into the upper troposphere and lower stratosphere (UTLS) are now available. HCl and HF were detected in the volcanic cloud from the Icelandic Hekla eruption in February 2000^34^. The observed halogen data were complemented by model estimates of injected bromine, which was not measured, but necessary to reproduce observed signals^35^. Furthermore, technical and scientific advances in satellite retrievals over the last 10 years has made it possible to detect halogen compounds in volcanic plumes of explosive eruptions reaching into the UTLS (Montserrat 2006, HCl^36^; Kasatochi 2008, BrO, and IO^37,38^; Sarychev 2009, HCl^39^; Puyehue-Cordón Caulle 2011, OClO, BrO, and HCl^40^; Nabro 2011, HCl^39^). Thus, an increasing number of small to large explosive volcanic events, spanning much of the planet, are now observed to inject halogens up to the UTLS and beyond (where available, estimated injection efficiencies from individual eruptions are in the range of ~2–5% (c.f. Table 1)).

Volcanic plume model simulations attempt to provide a basis for the discrepancy between the chlorine masses emitted from the volcanic vent and injected into the stratosphere. Early one-dimensional plume model studies of Plinian eruption columns indicated that less than 1% of emitted chlorine would reach the stratosphere; the rest was assumed to be scavenged by supercooled water^41^. A more recent plume model study, expanding to three dimensions and extending the microphysics of water in the plume to include the ice phase, simulates that 10–25% of emitted HCl is injected into the stratosphere^42^, since ice is much less efficient at scavenging chlorine than water droplets. Next, scavenging of HCl by ash has been found by laboratory experiments to not reduce the chlorine content of a large volcanic plume significantly^43^.

Because of the inconclusiveness of the early satellite-era observations of stratospheric halogen injection by volcanoes, this source has mostly been neglected^1,12,44^. Nevertheless, recent years have provided strong evidence that high amounts of chlorine and bromine up to hundreds of mega- and kilotons, respectively, can be released during large explosive eruptions^13,15,19,45–47^. From an average Central American Volcanic Arc (CAVA) eruption with a magnitude of 6, emissions up to ~30 megatons of chlorine and ~130 kilotons of bromine are supported by the evidence^15^. These eruptions have a recurrence time of approximately 130 years^14^. Bromine is much more effective for ozone destruction than chlorine^48^, ranging from ~65 to ~47 times more from present-day^49^ to future^50^ atmospheric conditions, depending on the background chlorine level^48^. Taking the combined chlorine and bromine from an average CAVA eruption into account, Krüger *et al*.^12^ estimated that even if as little as 10% of that amount is injected into the pre-ozone hole conditions stratosphere, the increase in halogen loading would be more than double the present day total anthropogenic background levels. For a future average CAVA eruption, the relative impact on stratospheric halogen loading would almost double again between present-day and 2100.

To study the potential atmospheric impacts of volcanic eruptions we use chemistry climate models. Getting reliable results from climate model simulations requires constraining the relevant parameters of the volcanic eruption. While satellite measurements provide the most appropriate atmospheric input parameters to evaluate volcanic impacts on the atmosphere and climate, very few (sub-)Plinian eruptions have occurred in the satellite era^39^. Due to the limited number of well-observed large volcanic events, one can use a much larger and extensive database of large to extremely large paleo-eruptions.

Such databases of volatile release can be assessed for a variety of paleo-eruptions where the amount of volcanic gases released is estimated employing the petrological method. By measuring pre-eruptive (in melt inclusions) and post-eruptive (in matrix glass) gas contents of the magma and using the resulting difference together with the erupted magma mass, one can calculate the minimum amount of different volatiles released during an eruption, neglecting the potential amount of volatiles released through the fluid phase, and from that specify the range injected to the stratosphere^12–14,29,47^. Due to analytical constraints, so far sulfur and chlorine has been the preferred target using the petrological method to determine gas emissions from large paleo-eruptions, but this is now changing, with more evidence on volcanic bromine injections to the UTLS becoming available.

A detailed dataset of degassing budgets of chlorine, bromine and sulfur from large paleo-eruptions of the CAVA is available covering the last 200 ka including 29 eruptions with eruption magnitudes (M_v_)^51^ from 4.5 to 8. The total erupted sulfur, chlorine and bromine masses range from 0.1 to 344 Mt, 0.17 to 807 Mt and 2.34 to 1100 kt, respectively^14,15^ (Fig. 1). The emissions of sulfur and halogens to the atmosphere from large to extremely large eruptions of the CAVA are well constrained with maximum errors between ±13% for chlorine, ±18% for sulfur and ±23% for bromine, over the period of 200 ka^13,15,52^. This new dataset facilitates constraining the volcanic impact on the atmosphere and environment through chemistry climate model simulations.

Only few modeling studies concerning stratospheric implications of volcanic halogens and aerosols from explosive volcanism are known to us. In a study of the Bronze-Age eruption of Santorini (~1600 BCE) petrologically determined chlorine and estimated bromine degassing budgets, with 2% halogen injection efficiency, were used as input for the 2D Chemical Transport Model (CTM) with simplified internally generated zonal mean winds^10^ (These degassing budgets for bromine have later been confirmed^28^). Simulations show peak ozone loss in the Northern Hemisphere (NH), ranging from 20–90% depending on the degassing budget scenario. Global ozone depletion reaches approximately 20% in the high volatiles scenario and the ozone layer takes about a decade to completely recover in this study. Similar to that, another 2D CTM study investigated volcanic eruptions under future climate emission scenarios co-injecting sulfur together with chlorine (and varying bromine background levels for sensitivity) of a Pinatubo strength eruption^11^, without considering volcanic bromine which is known to be highly variable for large eruptions^15^. This model study projects column ozone loss up to 4 years maximizing with 5% to 13% depending on the future anthropogenic chlorine level. These model results are, however, limited by the fact that they employ 2D CTMs with simplified mean atmospheric circulation^10^ or with prescribed meteorology focusing on the chemical response^11^. CTM studies with prescribed winds lack the complex and important interactive feedback through radiation on the atmospheric circulation and dynamics altering the transport and deposition of tracers and thus the composition of the atmosphere^6,30,31,53–56^.

In addition to the studies of explosive volcanism, a few 2D CTM and 3D General Circulation Model (GCM) studies of chlorine release from the Siberian Traps volcanism 250 Myrs ago^9,57^ are available. For extremely large continuous release of sulfur and chlorine, simulations show that the global ozone column decreased by up to 70%, a near-collapse of the ozone layer. The recovery of the ozone layer took approximately 10 years after the emissions have ended. Ozone depletion caused by halogen release from this type of volcanism is one of the hypothesized triggers for the Permian-Triassic boundary mass extinction.

In this paper we present unique results of the first complex interactively coupled chemistry climate model investigation of tropical sulfur- and chlorine- and bromine-rich explosive eruptions in the preindustrial atmosphere. We have used the high-top state-of-the art coupled chemistry climate model CESM1(WACCM)^58^ with volatile release data (S, Cl, Br) and sulfuric acid aerosols of an average CAVA eruption (average of 28 eruptions^14,15^; Fig. 1), a best possible estimate for representing large explosive eruptions on a global scale to date, as input. In addition to atmospheric radiation, temperature, circulation and composition changes, we also evaluate the potential impact of global ozone loss on the biosphere by calculating the change in biologically active UV radiation reaching the Earth´s surface after such an eruption. We will discuss our results in light of the existing literature, before giving a summary and perspectives for future studies.

## Results

### Ozone and stratospheric response of large tropical volcanic eruptions

In order to evaluate the effect that large Plinian eruptions have on the atmosphere and environment we have chosen the volatile mass of an average CAVA eruption as model input excluding the extremely large Los Chocoyos eruption^15^ (see Methods). Since the average consists of 28 eruptions of variable compositional signals (basaltic andesite to rhyolite)^15^ along an entire arc and a long-lasting time interval (200 ka) we consider it representative for the whole CAVA and also for large sulfur- and halogen-rich eruptions globally^14^ (Fig. 1, Table 1). We inject 10% of the erupted halogen mass (chlorine and bromine) into the stratosphere, a compromise given the large range and high uncertainties of injection efficiency estimates based on volcanic halogen observations in the UTLS^24,25,34–40^ and recent volcanic plume modelling^42^. We run three ensemble simulations, each with eight members and 12-years duration, one with volcanic halogen injections (**Halog**); one with volcanic sulfuric acid aerosol (surface area density, **SAD**); and one with both forcings applied (**Halog + SAD**). The initial conditions for the ensemble members were provided by branching from different years of the control simulation (additional details in Methods). The control simulation has constant 1850 forcings with duration of 30 years (**Ctr**). In addition, we performed a sensitivity simulation using 1% halogen injection efficiency and the same aerosol forcing (**1%Halog + SAD**). Table 2 gives a summary of the parameters used in the experimental setups.

**Table 2:** Summary of model simulations.

Volcanic forcing experiment	Sulfur aerosol forcing	Cl injection (Mt)	Br injection (kt)	Halogen injection location	Halogen Injection level	Injection date	Simulation length	No. ensemble members
Control (Ctr)	—	—	—	—	—	—	30 years	1
Halog	—	2.93	9.5	14°N, 89°W	29.7 hPa	January 1	12 years	8
SAD	Prescribed El Chichón^91^	—	—	—	—			
Halog + SAD	Prescribed El Chichón^91^	2.93	9.5	14°N, 89°W	29.7 hPa			
1%Halog + SAD	Prescribed El Chichón^91^	0.293	0.95	14°N, 89°W	29.7 hPa		8 years	1
HighHalogLev + SAD	Prescribed El Chichón^91^	Elevated surface emissions	Elevated surface emissions	—	—		10 years	1

In all simulations, background total stratospheric chlorine and bromine values are 0.47 ppbv and 5.68 ppt, respectively, originating from constant surface emissions of CH_3_Cl and CH_3_Br. These background values are consistent with previously reported pre-industrial background halogen levels^44^. The stratospheric Br values in our simulations may be too low (3–5 ppt) due to a lack of natural oceanic emissions of bromine compounds (Dorf *et al*.^59^ and updates), though there may be large uncertainties in the natural contribution of oceanic halogen emissions due to atmospheric and oceanic circulation changes (i.e., surface wind and sea surface temperature^60^) in a pre-industrial atmosphere. We addressed this issue through a sensitivity simulation applying increased background levels of 1.12 ppbv Cl and 16.99 pptv Br and the SAD volcanic forcing scenario (**HighHalogLev + SAD**).

The **Halog + SAD** simulations show that a stratospheric injection of volcanic chlorine and bromine leads to a global increase of the total halogen concentrations in the middle atmosphere lasting more than 7 years (Fig. S1). Partitioning between reactive (ClO_x_ and BrO_x_) and non-reactive compounds (HCl, HBr, ClONO_2_, BrONO_2_) varies with a larger proportion of Br in the form of reactive compounds (not shown). Global mean stratospheric concentrations of total Cl and Br during the first post-eruption year are 5.27 ppbv (>1000% increase) and 8.76 pptv (50% increase), respectively (Fig. S2). Present-day values in 2012 have reached about ~3.3 ppbv total Cl and ~20 pptv total Br^61^.

**Figure 2:**
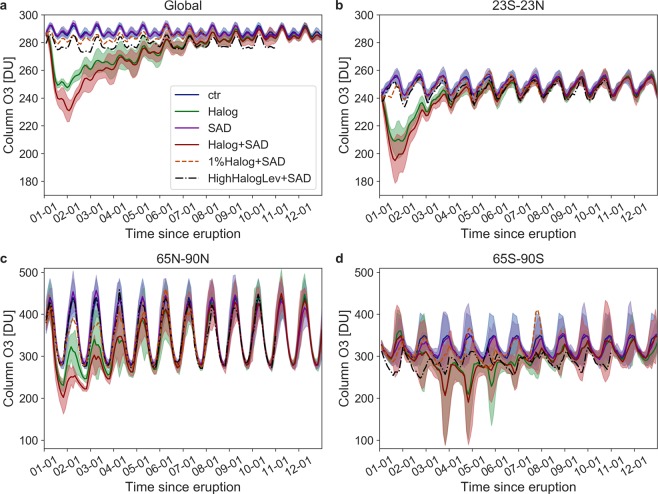
Column ozone response in [DU] for the different forcing experiments (see Table 2). Shown are the first 12 years after the eruption. Ensemble means (solid lines) and 2σ (shading). (**a**) Global; (**b**) Tropics; (**c**) Arctic; (**d**) Antarctic.

In response to the increase in halogen and sulfuric acid aerosol concentrations, **Halog + SAD** globally averaged ozone levels drop to a minimum below 240 DU, a 20% decrease in global mean ozone column, during the first 18 months after the eruption (Fig. 2a). Global ozone stays significantly below climatological levels for 10 years. The **Halog** ensemble shows a smaller ozone response than **Halog + SAD**. The difference between the two scenarios becomes markedly smaller after the first 3 years, when the SAD forcing has decayed. In the **SAD** experiment the global ozone response shows a small increase, caused by increased heterogeneous chemistry, which reduces the concentrations of ozone-destroying NO_x_^33^, and by radiative-dynamical effects^62^.

These results are sensitive to the halogen injection efficiency. In the **1%Halog + SAD** sensitivity simulation the mean increase of total Cl and Br over the first post-eruption year is 213% and 6% (Fig. S2). This translates to a small decrease in column ozone (~5%), only slightly outside the natural variability of the control run (Fig. 2). In the **HighHalogLev + SAD** sensitivity, increasing the background halogen concentration leads to lower global ozone levels as expected, while there is no clear ozone response to the SAD forcing (Fig. 2). In the following, we will focus on the ensemble mean of the **Halog + SAD** runs when discussing regional responses and surface impacts.

**Figure 3:**
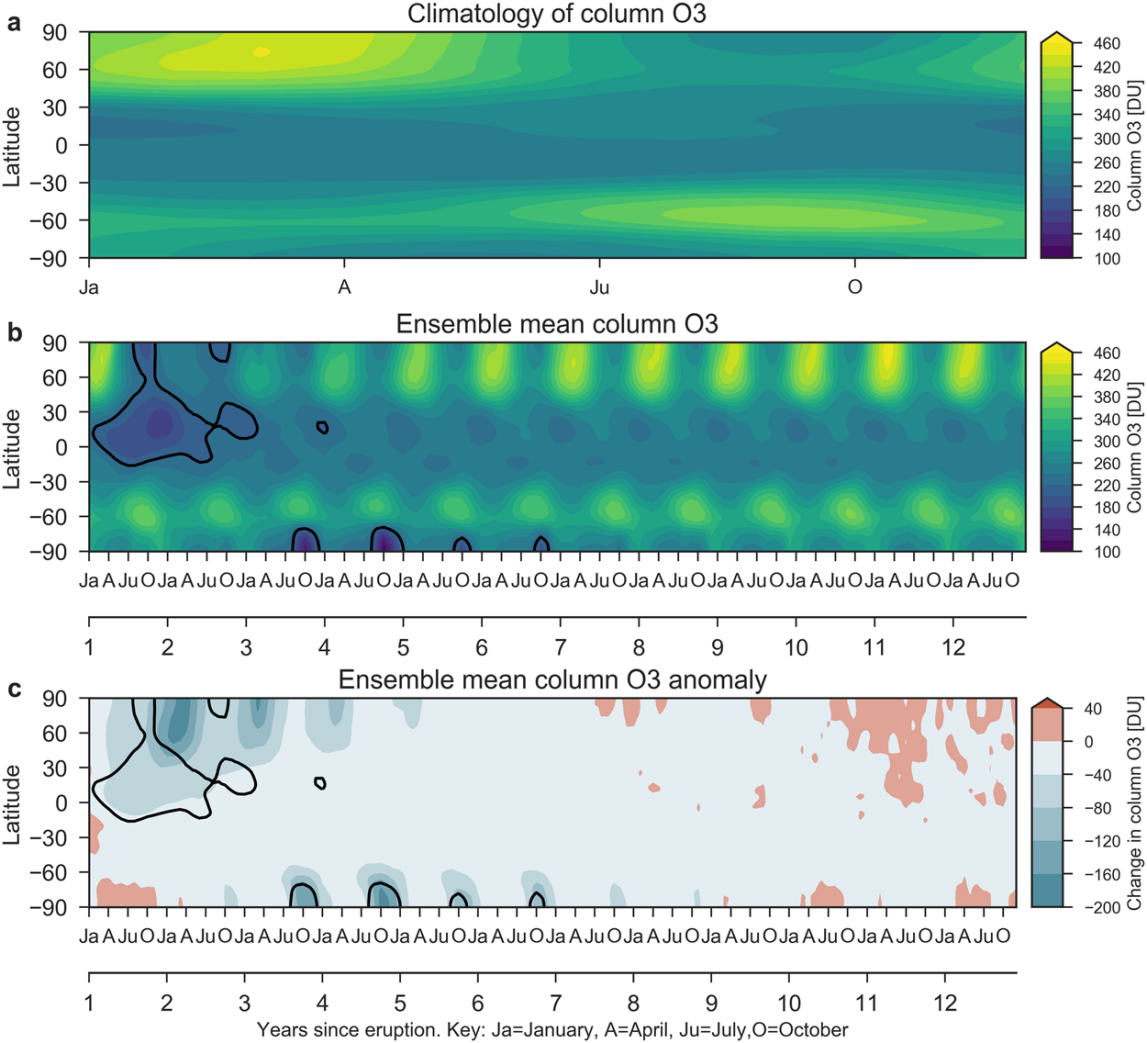
Ensemble mean zonal mean ozone response to: (**a**) Climatological annual cycle of the column ozone from the control run. (**b**) Column ozone and (**c**) column ozone anomalies over the first 12 years after the eruption in the Halog + SAD forcing experiment. The black contour in (**b**,**c**) marks the region where column ozone drops below 220 DU which is the definition of the Antarctic ozone hole.

**Figure 4:**
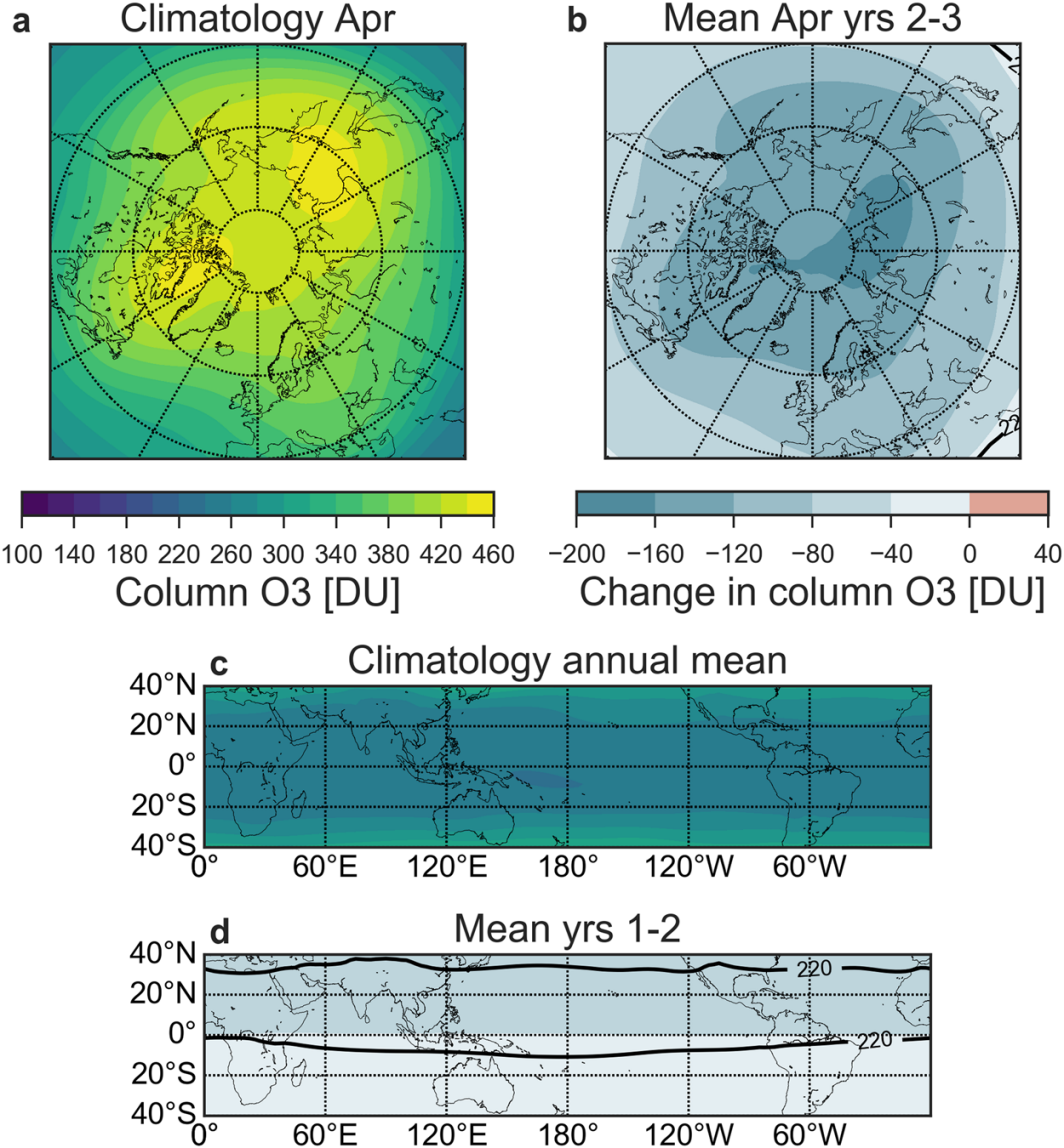
Ensemble mean Arctic (**a**,**b**) and Tropical (c-d) ozone response to the Halog + SAD forcing experiment. (**a**) April climatological column ozone. (**b**) Column ozone anomalies averaged over April of post-eruption years 2–3. (**c**) Annual mean climatological ozone. (**d**) Anomalies averaged over post-eruption year 1 and 2.

**Figure 5:**
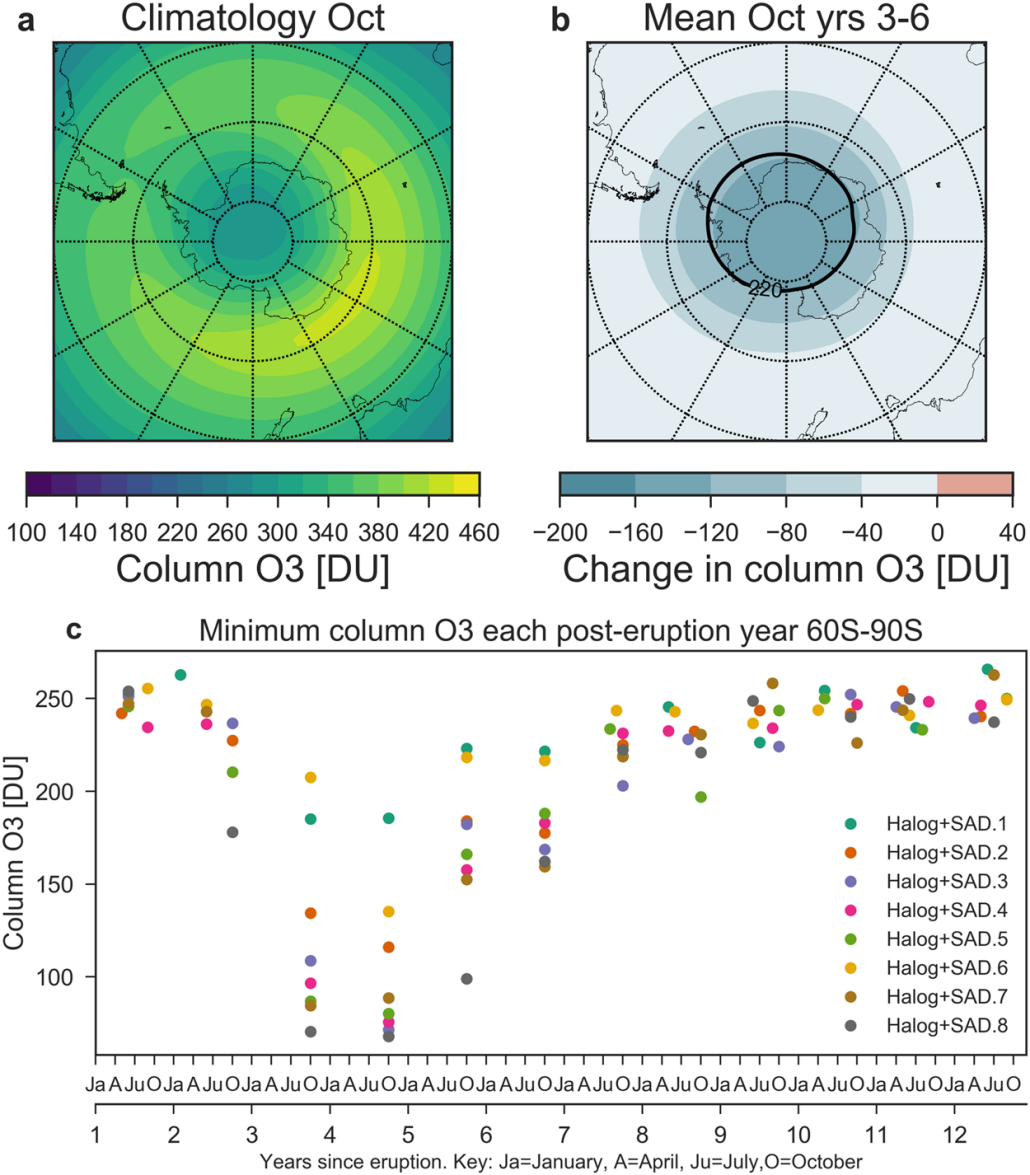
Antarctic ozone response to the Halog + SAD forcing experiment. (**a**) Antarctic climatological ozone for October. (**b**) Ensemble mean ozone anomalies for October, averaged over post-eruption years 3–6. (**c**) Annual Antarctic minimum column ozone for each ensemble member of the Halog + SAD experiment in the first 12 years after the eruption.

The atmospheric response to the CAVA eruptions follows a distinct spatio-temporal evolution (Figs 2 and 3). The immediate response is in the tropics and middle latitudes on the NH, with column ozone dropping below 220 DU (with minimum below 200 DU) during the first two years. In the tropics, this corresponds to 10–20% ozone depletion, which is far outside the small tropical natural ozone variability. In the first autumn after the eruption, the Arctic minimum column ozone value of 200 DU occurs, while the maximum decline (45%, >200 DU) takes place in the following spring. Analyzing the maximum spring depletion events of the post-eruption years 2 and 3 reveals average ozone decrease of more than 120 DU (20%) over the polar cap, reaching as far south as 50° N over certain regions (Fig. 4). In the Antarctic, a strong response is simulated during austral spring starting in the 3rd simulation year (Figs 2 and 3). Depletions below 220 DU occur over large parts of the Antarctic continent, similar to a present-day ozone hole (Fig. 5). This phenomenon appears during simulation years 3–6 with the minimum column ozone below 100 DU and maximum ozone hole area extent in October of simulation year 4 (not shown).

There is a large spread in the minimum Antarctic column ozone values between the model ensemble members, which corresponds to the transport of chlorine and bromine reservoir compounds to the Southern Hemisphere (SH) during the first post-eruption year (Fig. S3). The intra-ensemble spread of the transport to the SH is large even though the models’ Quasi Biennial Oscillation is fixed in the easterly phase and sea surface temperatures are held constant, i.e., there is no El Niño Southern Oscillation variability. By post-eruption year 4 this translates into an intra-ensemble spread of the average SH concentration of stratospheric halogen of more than a factor of 2 between the lowest and highest ensemble members (Fig. S3c). During Antarctic ozone depletion, heterogeneous chemistry on Polar Stratospheric Clouds (PSCs) is an important reaction for halogen radicals. The temporal evolution of PSC formation, halogen activation and subsequent denitrification of the Antarctic stratosphere closely follows observed present day ozone depletion (not shown). In the Arctic, in contrast, PSC formation is almost completely suppressed during post-eruption year 1, only re-emerging the following winter.

**Figure 6:**
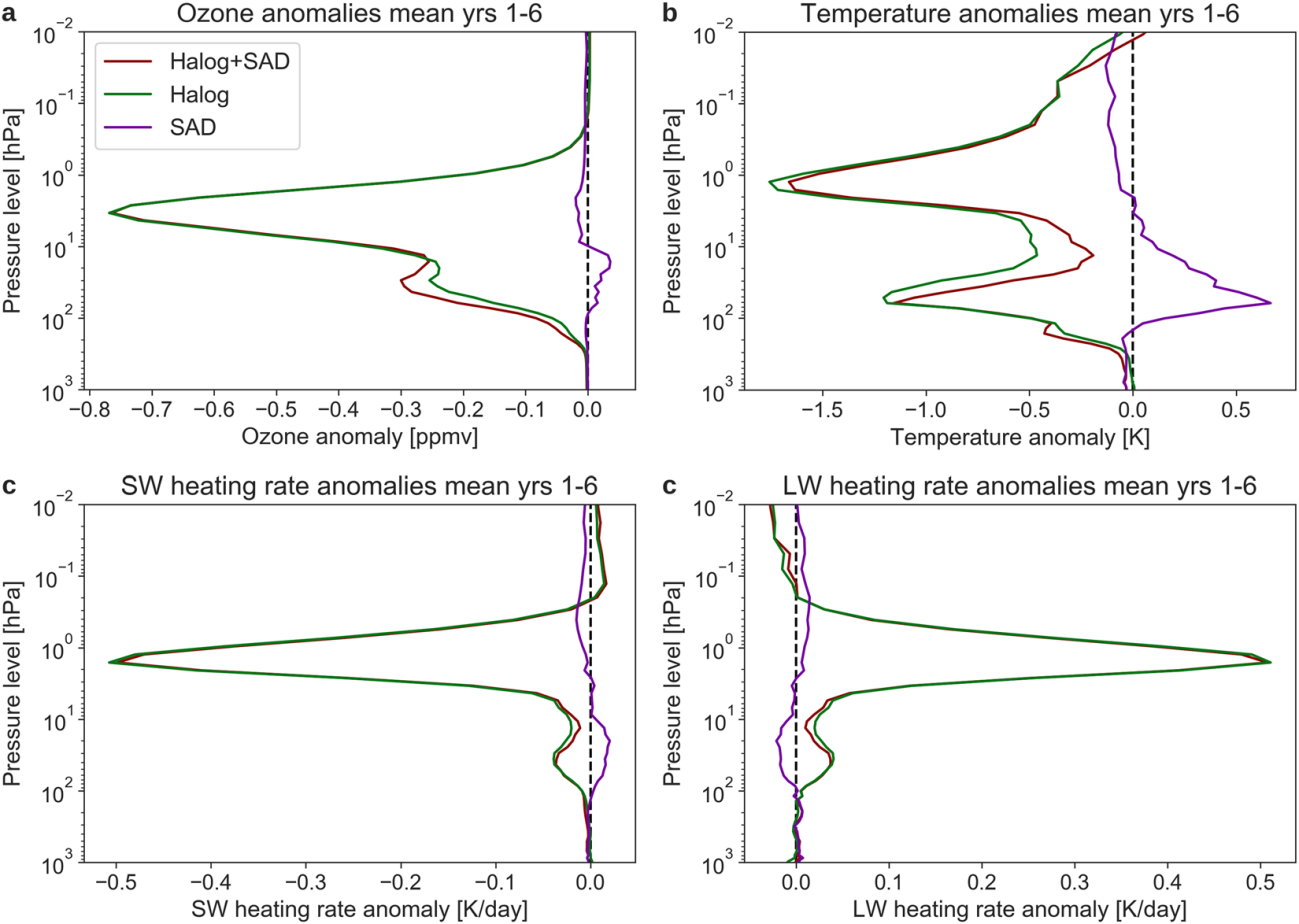
Global mean profiles averaged over post-eruption years 1–6 for the three forcing experiments. (**a**) Ozone concentration anomalies. (**b**) Temperature anomalies. Short wave (**c**) and long wave (**d**) heating rate anomalies.

Profiles of global mean temperature anomalies averaged over the first six post-eruption years indicate that the stratospheric response to the **Halog + SAD** forcing is distinct cooling at two layers (60 and 1 hPa) in the lower and upper stratosphere (Fig. 6). (Lower stratosphere: 100–30 hPa (~16 to 24 km altitude); Upper stratosphere: 5–1 hPa (~35–50 km altitude)). Initially, the cooling is strongest in the lower stratosphere around 30 hPa (not shown). After simulation year 2 the maximum cooling of more than 3 K occurs in the upper stratosphere. The model experiments reveal that the ozone depletion by Cl and Br cools the stratosphere at these two distinct layers (60 hPa and 1–2 hPa) while in contrast the SAD enhancement causes warming in the lower stratosphere (around 50 hPa). As a consequence, the stratospheric cooling is most pronounced in the Halog experiment (Fig. 6). Cooling of the lower stratosphere is much more pronounced in the NH and in the first two years post eruption, while the upper stratosphere cools globally with peak cooling in the third year after eruption.

**Figure 7:**
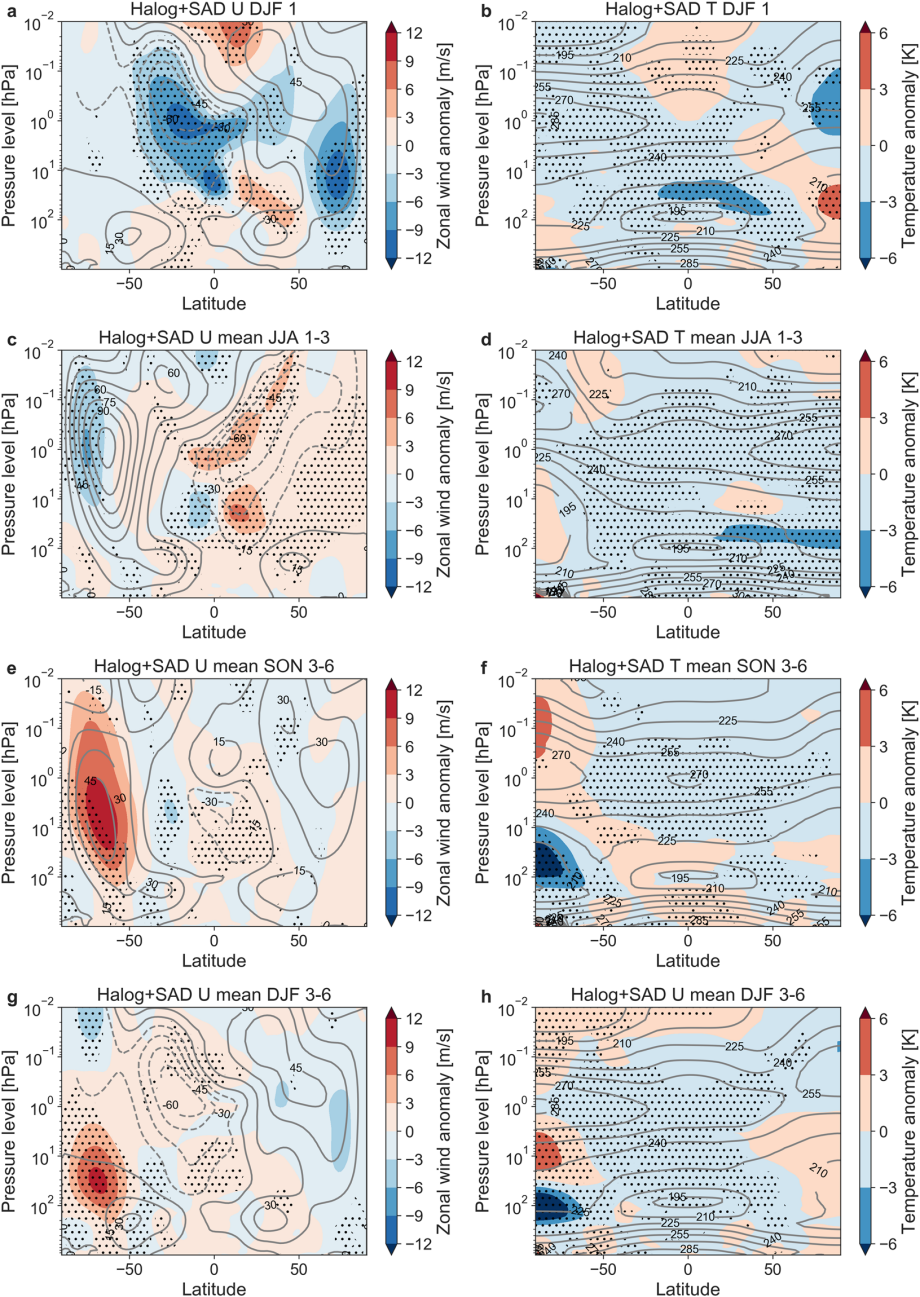
Ensemble mean zonal wind and temperature response to the Halog + SAD forcing experiment during: (**a**,**b**) First post-eruption NH winter (DJF). (**c**,**d**) Averaged over NH summer of post-eruption years 1–3. (**e**,**f**) Averaged over SH spring (SON) of post-eruption year 3–6. (**g**,**h**) Averaged over SH summer (DJF) of post-eruption years 3–6. Gray contours represents the Control climatology of zonal wind (temperature) with intervals of 15 m/s (K). Stipling indicates changes at the 95% confidence level.

The zonal wind response of WACCM to the combined volcanic forcing of the first post-eruption NH winter (DJF (December, January, February)) is a weakening of the westerly wind in the stratosphere (Fig. 7a) as the temperature gradient between the tropics and the NH high latitude is reduced (Fig. 7b). Ozone depletion in the tropical stratosphere causes reduced shortwave warming while depletion in the polar night causes warming by reducing the longwave cooling (not shown). The changes in zonal wind and temperature are significant at the 95% confidence level. The corresponding response for a typical sulfur-rich tropical eruption is a strengthening of the stratospheric westerlies of the first winter, caused by an increased meridional temperature gradient^63^. Due to enhanced ozone depletion and strengthening of the polar vortex in Arctic spring (Figs 3 and 4), NH summer (JJA (June, July, August)) of post-eruption years 1–3 reveal positive zonal wind anomalies all the way to the surface, causing a positive phase of the Northern Annular Mode (Fig. 7c,d). (The Annular Modes are atmospheric circulation patterns with lower than normal pressures over the polar region and stronger surrounding westerly winds along ~60°)^64^.

The SH winter (JJA) in post-eruption years 1–3 shows the same pattern as for the first NH winter, with a weakened polar vortex (Fig. 7c,d). During the averaged Antarctic ozone hole events of the post-eruption years 3–6 (Fig. 5), the polar vortex is strengthened from the upper mesosphere to the stratosphere during austral spring (SON (September, October, November)) (Fig. 7e,f) and migrating downwards to the lower stratosphere during DJF (Fig. 7g,h). During both seasons, SON and DJF, the significant increase in westerly wind reaches all the way down to the surface, leading to a positive phase of the Southern Annular Mode, similar as observed for present-day ozone hole conditions^65^.

The atmospheric response in WACCM to our sulfur- and halogen-rich eruption deviates markedly from the well-known response to sulfur-rich, but halogen-poor eruptions like Pinatubo. In general, tropical sulfur-rich Plinian eruptions heat the tropical stratosphere and cool the polar stratosphere leading to an increase in the equator-pole temperature gradient resulting in increased strength of the polar vortices, particularly during the first post-eruption winter of the NH^54,63,66,67^. Our simulations highlight that with strong sulfur and halogen injections, the atmospheric circulation pattern is reversed in the first post eruption NH winter. The ozone depletion causes cooling of the tropical stratosphere and a reduced meridional temperature gradient resulting in a weaker and warmer polar vortex and strong ozone loss compared to the following 2^nd^ and 3^rd^ winters.

### Modeled effect on biologically active UV

**Figure 8:**
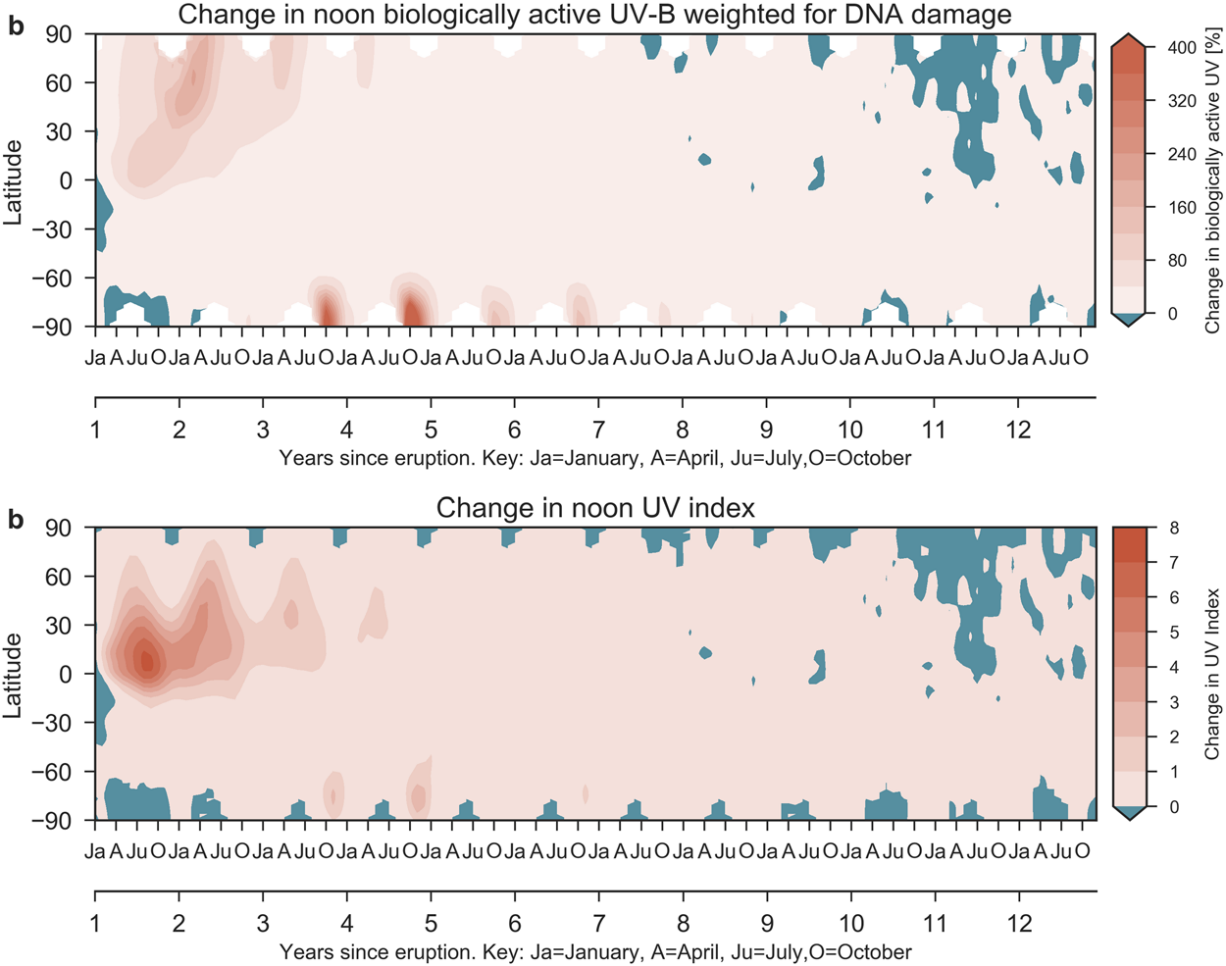
Ensemble mean surface UV change during the Halog + SAD forcing experiment after the first 12 years of the eruption. (**a**) Relative change in bioactive UV-B weighted for generalized DNA damage. (**b**) Change in UV index. White regions indicate polar night.

We have used the radiation transport model TUV^68^ to calculate the change in surface biologically active ultraviolet B (UV-B) radiation and the UV index (see Methods). Globally, the clear sky UV-B increases by a maximum of 80% in the tropics, averaging to above 40% over the first two years (Fig. 8a). The NH poleward of 30 N experiences more than 190% peak increase in UV radiation in the first two years after the eruption, increasing even more in parts of the Arctic (Fig. 8a). During maximum Antarctic ozone depletion, the increase in biologically active UV exceeds 400%.

To quantify the impact of the ozone depletion on human populations we have calculated the change in the clear sky ultraviolet index (UVI), which is a standard measure of the strength of sunburn-causing ultraviolet radiation (see Methods). Figure 8b shows the evolution of the zonal mean, monthly mean anomaly of clear-sky UVI at noon for all latitudes. The maximum increase in UVI is more than 7 units in the NH tropics and subtropics. Absolute zonal mean tropical UVI values peak above 22, which is similar to present-day global UV maximum found at high altitudes in the tropics. NH midlatitude UVI increase peaks at 4 units during the summers of the first two post-eruption years. Much of the midlatitudes experiences UVI above 15, which is similar to pre-eruption and present-day values in the tropics. All of the NH has maximum increases in UVI of more than 50%. Because of the low amount of UV radiation over the Antarctic, the peak UVI increase is only 2 units during the active ozone hole period. Still, this represents more than a doubling of weighted UV-B flux at the surface.

## Discussion

Our coupled chemistry climate model results show that the global atmosphere will be strongly impacted over a timescale of ~10 years by a tropical sulfur and halogen-rich volcanic eruption of a magnitude similar to an average CAVA eruption. This is a much longer-lasting impact than the well-known and reported one from a sulfur-rich, but halogen-poor eruption of similar size^4,5,11^. A key difference is that, while sulfuric acid aerosols typically fall out of the atmosphere within the first 3 years^4^, the halogens being in the gas phase are mixed throughout the stratosphere. Thus, the lifetime of volcanic halogens in the stratosphere is dominated by their chemical life cycle and the stratospheric circulation time scale.

This study and Cadoux *et al*.^10^, using different models and different volcanic eruptions (see Table 1), come to the following similar conclusions which may be a bit surprising: high halogen loading in the stratosphere lasting up to 10 years, global ozone depletion of ≥20% following the eruption which is strongest in the 2–3 years post-eruption at NH mid-latitudes, and ozone recovery in about a decade. Cadoux *et al*.^10^ takes a stronger volcanic eruption (M_v_ = 7) at NH mid-latitudes into account but a smaller halogen injection efficiency of 2% whereas we simulate a weaker eruption (M_v_ = 6.4) with a higher halogens injection efficiency of 10% in the tropics. These different model experiment set-up details (see Table 1) may explain the similar global ozone impact as well as our larger ozone impact in the SH, as can be expected from the transport of a volcanic cloud by the atmospheric general circulation for a tropical versus an extra-tropical eruption^30,69^.

If we take such an average CAVA eruption scenario into account for a pre-industrial or future climate atmosphere with a low chlorine background, several impacts are obvious: Ozone column values that today are only associated with the Antarctic ozone hole would occur over large parts of the NH, and even prevail in the tropics for two full years, and an Antarctic ozone hole would appear for several years.

Unlike the current anthropogenic ozone depletion, which includes only small changes in the tropics and moderate changes in the mid-latitudes, the resulting increase in ultraviolet radiation due to volcanic ozone depletion is large also over populated and biologically important areas. This difference in behavior is caused by the different sources of halogen radicals from anthropogenic and volcanogenic halogens. While most anthropogenic halogenated compounds (such as chlorofluorocarbons) have to be transported to the upper stratosphere before being activated by UV light, volcanic halogens can be activated immediately upon injection into the stratosphere, thus causing larger impacts in the tropics and mid-latitudes (depending on the location of the eruption). Human populations in these areas would experience increased risk of sunburn and DNA damage. The volcanic increase in UV could impact plants and agriculture in detectable ways. Several crops are sensitive to changes in UV exposure, leading to decreased yields^70,71^ in addition to terrestrial^72^ and marine^73^ ecosystems impacts. Marine photosynthetic productivity is highly sensitive to UV exposure, reducing primary productivity in the ocean^73–75^. Present-day global ozone depletion of 16% (which is similar to the 20% depletion we find) has been estimated to reduce phytoplankton productivity by 5%, with possible losses of up to 7% of fish harvests as a consequence^76^.

The impacts of halogen rich volcanism through increases in surface UV radiation has previously been evaluated for the Siberian Traps Large Igneous Province volcanism using a simplified analytical formula^77^ relating column ozone to changes in UV flux at the surface^9^. The reported global impacts of >1000% increase in surface UV-B over much of the planet originates from the enormous size of the eruptive activity^9^. This type of eruptive activity (long-lasting, mainly non-explosive) is, however, very different from the highly explosive volcanism of the Central American Volcanic Arc.

Given a proxy for past UV radiation with sufficient resolution, such as a well-preserved sediment core containing plant spores or pollen or a high-resolution ice core, the signal from a large sulfur- and halogen-rich eruption like the one we have simulated should be detectable. Indeed, methods for detecting changes in UV radiation using plant spores or pollen have been developed^78,79^, though they have not been applied on high resolution archives far back in time yet. A recent ice core-based study reported potential paleo ozone depletion caused by volcanic halogen^80^. A 192-year series of sulfur- and halogen-rich eruptions on the Antarctic continent was detected to cause increased ultraviolet irradiance at the surface, altering the sulfur isotope ratios and the concentration of bromine in the preserved ice cores. This long period of increased volcanic activity coincided with abrupt warming of the Antarctic continent, consistent with the response to a long-term ozone depletion event^81,82^. To detect a single volcanic event, in contrast to a series of eruptions occurring over decades, the resolution of the proxies has to be annual or at least sub-decadal. For ice cores, annual resolution is achieved for more recent time periods^83^.

## Summary

Our study highlights that representative volcanic eruptions from the tropics, e.g. Central American Volcanic Arc, had and have the potential to deplete ozone by ~20% globally, causing Antarctic ozone hole conditions across the NH, the tropics and the Antarctic. In our simulations, strong impacts on stratospheric composition, temperature and circulation were found on the pre-industrial atmosphere. Contingent on the assumptions made on eruption location, halogen content and injection efficiency we find that the ozone layer takes up to 10 years to fully recover to pre-eruption conditions confirming previous studies. The inclusion of volcanic halogens in addition to sulfur changes the stratospheric impact of the eruption from a warming to a cooling. In addition, the NH polar vortex is weakened instead of strengthened in the first post-eruption winter. Following, severe ozone depletion over the polar regions causes strengthening of the polar vortices and positive annular mode circulation in years 1–3 in the NH and in years 3–6 in the SH.

The depletion of ozone causes large increases of ultraviolet radiation at the surface over the entire globe with >60% peak increase (NH 40–190%, tropics ~40% and Antarctic >400%) with potential consequences for crop plants, marine life as well as human populations during that period. These results may impact upcoming studies on high-resolution proxy ice cores and archives of plant spores and pollen to detect volcanically induced paleo ozone holes of the kind we project in our simulations. The methodology seems to be available, if a suitable high-resolution proxy archive is accessible. Several of the large to extremely large eruptions of the CAVA time series are potential targets for such future investigations.

## Methods

### CESM1(WACCM)

In the present study, the Community Earth System Model, Version 1 (CESM1) the Whole Atmosphere Community Climate Model (WACCM)^58^, based on the Community Atmospheric Model Version 4 is used. We refer to this setup as CESM1(WACCM), a comprehensive coupled chemistry climate model spanning the whole atmosphere from the surface to the lower thermosphere with model top at ~140 km altitude. The chemistry module in CESM1(WACCM) includes the O_x_, NO_x_, HO_x_, ClO_x_ and BrO_x_ chemical families, implementing 52 compounds, 127 gas-phase reactions, 48 photolytic reactions and 17 heterogeneous reactions on three types of aerosols^84^. Polar stratospheric clouds are formed interactively in the model, and stratospheric sulfuric acid aerosols are included as prescribed surface area density (SAD) files^85^. CESM1(WACCM), as a coupled chemistry climate model allows us to explore the coupling between radiation, temperature, circulation, chemistry and composition in the atmosphere. The horizontal resolution is 1.9° longitude by 2.5° latitude with 66 hybrid sigma pressure layers from 992 to 5.5e-6 hPa. The quasi-biennial oscillation is prescribed and fixed in the easterly phase. Sea surface temperatures are prescribed to preindustrial (1850) conditions.

### Model forcing data

Input data for the coupled chemistry climate modeling is based on our own published data from measurements of the CAVA material, comprising melt inclusions and degassed matrix glasses from basaltic-andesitic to rhyolitic tephras from Plinian eruptions^13–15^. These input data are based on a three-stage procedure where in a first step the eruptive masses of single eruptions were determined using a) tephra mapping (thicknesses and distribution) and b) volume and subsequent mass calculations by including rock densities. The latter follows the exponential decay approach^14,15,86–90^. For the second step volatiles (S, Cl, Br) were measured in tephra samples for each eruption using electron microprobe (S, Cl) S) and synchrotron XRF (Br). We use the conventional petrological method where pre-eruptive volatile contents given in melt inclusions of host minerals subtracted by post-eruptive volatile contents given in erupted and degassed glasses give the degassed fraction of volatiles. Knowing the total eruptive mass from step 1 gives the volatile release during the eruption available for atmospheric injection^13–15^. In the last and third step, we apply a 90% reduction^12,42^ of the halogen volatiles, due to effective scavenging on the way into the stratosphere to derive representative stratospheric injection parameters for the chemistry climate modeling.

Based on the available data from the CAVA^14,15^, we used an average of 28 eruptions, excluding the extraordinarily large Los Chocoyos eruption, as input parameter for the coupled chemistry climate modeling. These average CAVA data represent a large sulfur- and halogen-rich eruption and are compared to the largest eruptions of the “satellite era”, the Pinatubo, El Chichón eruptions as well as other eruptions (Table 1). Degassed volatiles are, where available, presented as both petrological estimates as well as satellite estimates to emphasize the large uncertainties of both methods.

Whereas the satellite data are limited to eruptions in the last three decades the petrological method has the advantage of targeting paleo volcanic eruptions of known geographic sources. Additionally, the bromine releases used are based on measurements in contrast to studies using less well-constrained indirect estimations from element ratios (e.g. Cl/Br, S/Br^10,47^) that are prone to high, and unsystematic variability due to pre-eruptive fluid partitioning^15^.

Nevertheless, the petrological method might underestimate the volatile release in certain cases, since some volatile species (e.g. S, Br) may strongly be affected by pre-eruptive, magma composition-dependent fluid partitioning, and not be covered by measurements^14,15^, even though the fluid phase contributes to injected volatiles. This may lead to the petrological method underestimating the volatile release of an eruption by a factor of 10 for SO_2_^29^ and a factor of 2 or more for halogens^15^.

Since CESM1(WACCM) handles stratospheric sulfuric acid aerosols through prescribed surface area density (SAD) fields, we compare the petrological sulfur estimate of the average CAVA eruption to major present-day eruptions (Table 1). For the average CAVA the petrological estimate of erupted sulfur mass is 5.7 Mt which is in the range of satellite estimate of stratospheric sulfur injection from the 1982 El Chichón eruption (7.5 Mt). Therefore, we adapted the standard El Chichón SAD forcing^91^ for CESM1(WACCM) for our simulations by moving the March/April eruption to January (Fig. S5).

### Model setup and experiments

We performed a 30-year control simulation (called **Ctr**) with constant pre-industrial (1850) atmospheric conditions and sea-surface temperatures and three different forcing experiments: (1) Increased stratospheric aerosol SAD using the El Chichón 1982 volcanic forcing^91^ described above (called **SAD**). (2) Chlorine and bromine injections representing an average CAVA eruption (called **Halog**). The halogens were injected into four grid cells (12.32°-16.10° N, 86.5°-91.5° W) at 29 hPa (approximately 24 km), which is roughly consistent with the estimated height of the SO_2_ injection observed for Pinatubo^92^. (3) Combined SAD forcing and halogen injections (called **Halog + SAD**). The eruption date is set to January 1. In each of the experiments we performed eight ensemble simulations branched from different Januaries of the control run. In all three forcing scenarios the same initial conditions were used for the eight ensemble members and all model experiments were run for 12 years. In addition two sensitivity studies were performed. A single simulation with 1% halogen injection efficiency (**1%Halog + SAD**) and a simulation of the impact of the SAD forcing under increased background halogen levels (**HighHalogLev + SAD**).

### UV calculations using the tropospheric ultraviolet and visible (TUV) radiation model

Calculations of UV radiation at the surface were carried out using the Tropospheric Ultraviolet and Visible (TUV) radiation transport model^68^. In our setup TUV solves the radiative transfer equations given the parameters: date of the year, position, time of day, column ozone values and total aerosol optical depth at 550 nm to take aerosol scattering into account. We use zonal mean ozone column and aerosol optical depth fields to calculate the zonal mean UV flux and Ultraviolet Index (UVI) for both the climatology and the ensemble mean of the full forcing experiment at noon. From this we calculate the change in UVI and UV flux.

### Statistical significance testing

Statistical significance was calculated with the scipy.stats module in Python. To test statistical significance of global and regional ozone changes we applied the Student’s t-test on the individual samples, allowing the distributions to have different variances. For calculating significances of zonal mean anomalies of zonal wind and temperature we used the Kolmogorov-Smirnov test as the data were not normally distributed. By concatenating the ensemble members of the experiment and the individual years of the control run along a record dimension, we could treat each latitude and level index separately for testing. We then tested whether the experiment ensemble differed significantly from the control at each index. This way we could verify where the experiment response was different from the natural variability of the control simulation.

## Supplementary information

Supplementary material

## Data Availability

All simulation data will be archived in the NIRD Research Data Archive on acceptance of the manuscript. Post processing and visualization of data was performed with Python and the code and post processed data files are available on request from the corresponding author.

## Code Availability

The CESM1 model and the radiation transport model TUV are developed and supported by the National Center for Atmospheric Research (NCAR) and are publicly available.
