# Supplementary material for: Global ozone depletion and increase of UV radiation caused by pre-industrial tropical volcanic eruptions
Source: Sci Rep. 2019 Jul 1;9:9435. doi: 10.1038/s41598-019-45630-0 (PMC6603163; doi:10.1038/s41598-019-45630-0)
Supplement: Supplementary file 1 — Supplementary material [file 41598_2019_45630_MOESM1_ESM.pdf]

Global ozone depletion and increase of UV radiation caused by pre-industrial tropical volcanic eruptions

Hans Brenna, Steffen Kutterolf, Kirstin Krüger*

*Correspondence to kirstin.kruger@geo.uio.no

Affiliations
Hans Brenna, Kirstin Krüger (corresponding author)

Section for Meteorology and Oceanography, Department of Geosciences, University of Oslo, P.O.

Box 1022 Blindern, 0315 Oslo, Norway

Steffen Kutterolf

GEOMAR | Helmholtz Centre for Ocean Research Kiel, Wischhofstrasse 1-3, 24148 Kiel, Germany

Supplementary material

Hans Brenna, Steffen Kutterolf, Kirstin Krüger

1 HCl and HBr

Hydrogen chloride (HCl) and hydrogen bromide (HBr) were injected for the average CAVA eruptions. Figure S1 shows the time evolution of the global concentration of HCl and HBr post-eruption.

2 Stratospheric Cl and Br

We have calculated the average stratospheric concentration of total inorganic chlorine (Cl_y) and bromine (Br_y). In Figure S2 we show the temporal evolution of the concentration of Cl_y, Br_y in our simulations.

3 Hemispheric asymmetry

In the model simulations we found a large intra-ensemble spread in the severity of the simulated ozone depletion. To understand this spread we calculated the average stratospheric inorganic chlorine (Cl_y) concentration on each hemisphere over time and the ratio between them (Figure S3). There is an approximately linear relationship between the annual mean SH hemispheric mean Cl_y and column ozone (Figure S3). The corresponding relationships for Br_y is qualitatively the same as for Cl_y and thus not shown.

4 Zonal wind and temperature response during the first post-eruption DJF. Comparison of Halog+SAD and SAD

We compare the ensemble mean zonal mean response to the Halog+SAD and SAD forcing experiments during the first post-eruption DJF in Figure S4.

5 Figures

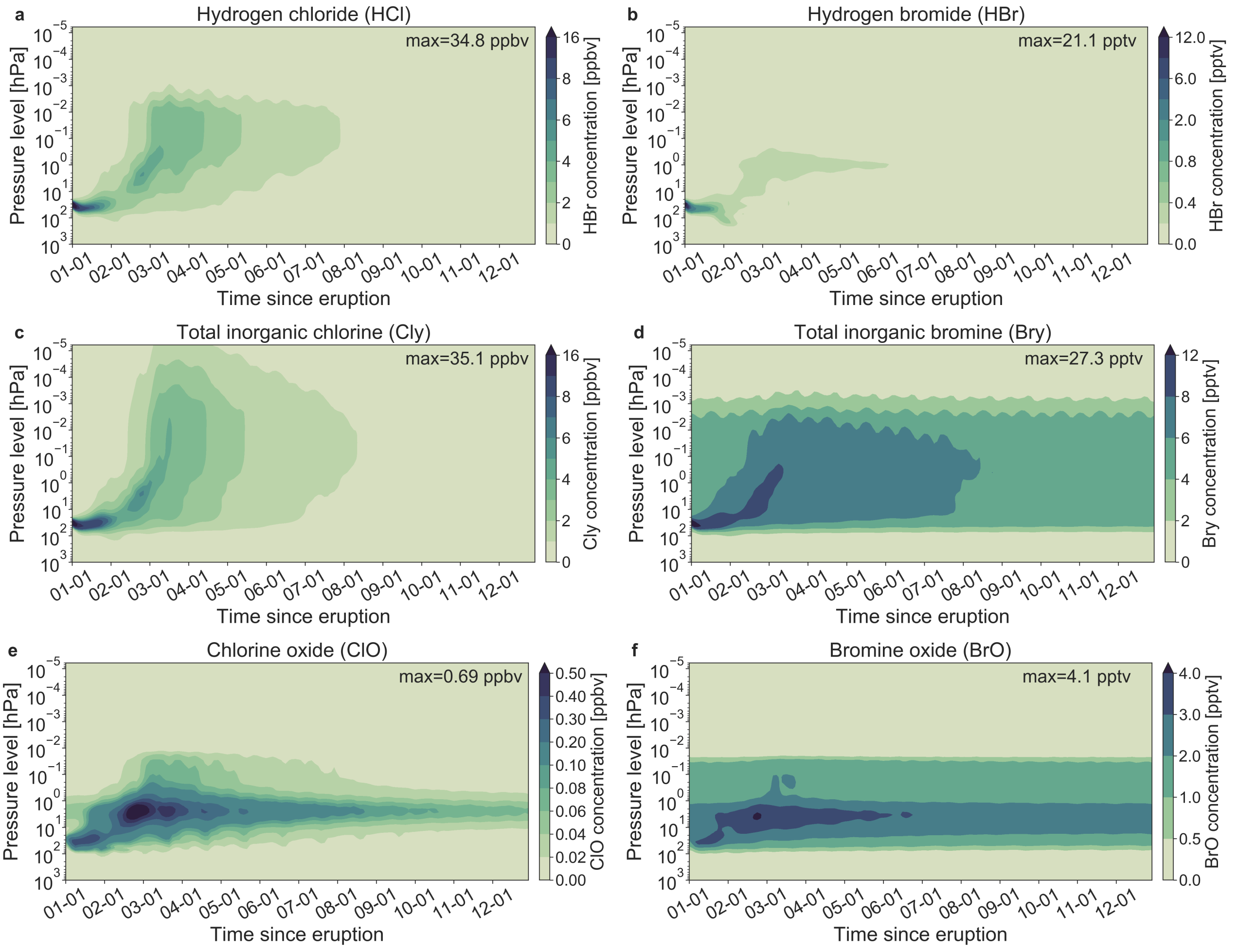

Figure S1: Global spread of injected halogens (volume mixing ratio). The halogens were injected as hydrogen chlorine (HCl, a) and hydrogen bromine (HBr, b). In addition we show the total inorganic chlorine (Cl_y, c), bromine (Br_y, d) and the reactive compounds ClO (e) and BrO (f). The global mean maximum is written in each panel.

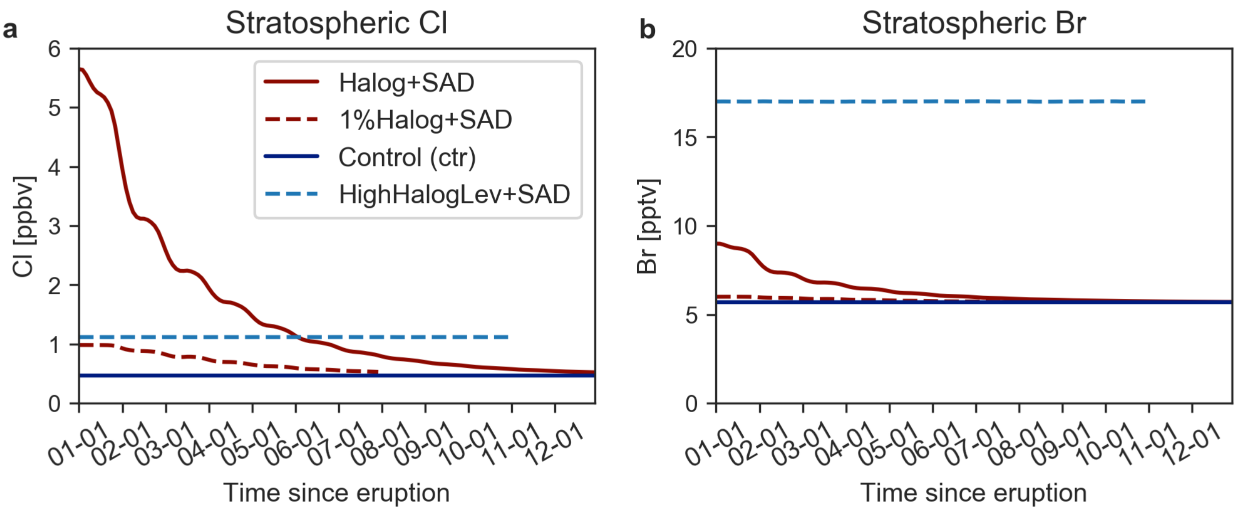

Figure S2: Average stratospheric concentration of total inorganic chlorine (Cly, a), bromine (Bry, b) for the control (ctr), the Halog+SAD, 1%Halog+SAD and HighHalogLev+SAD forcing experiments.

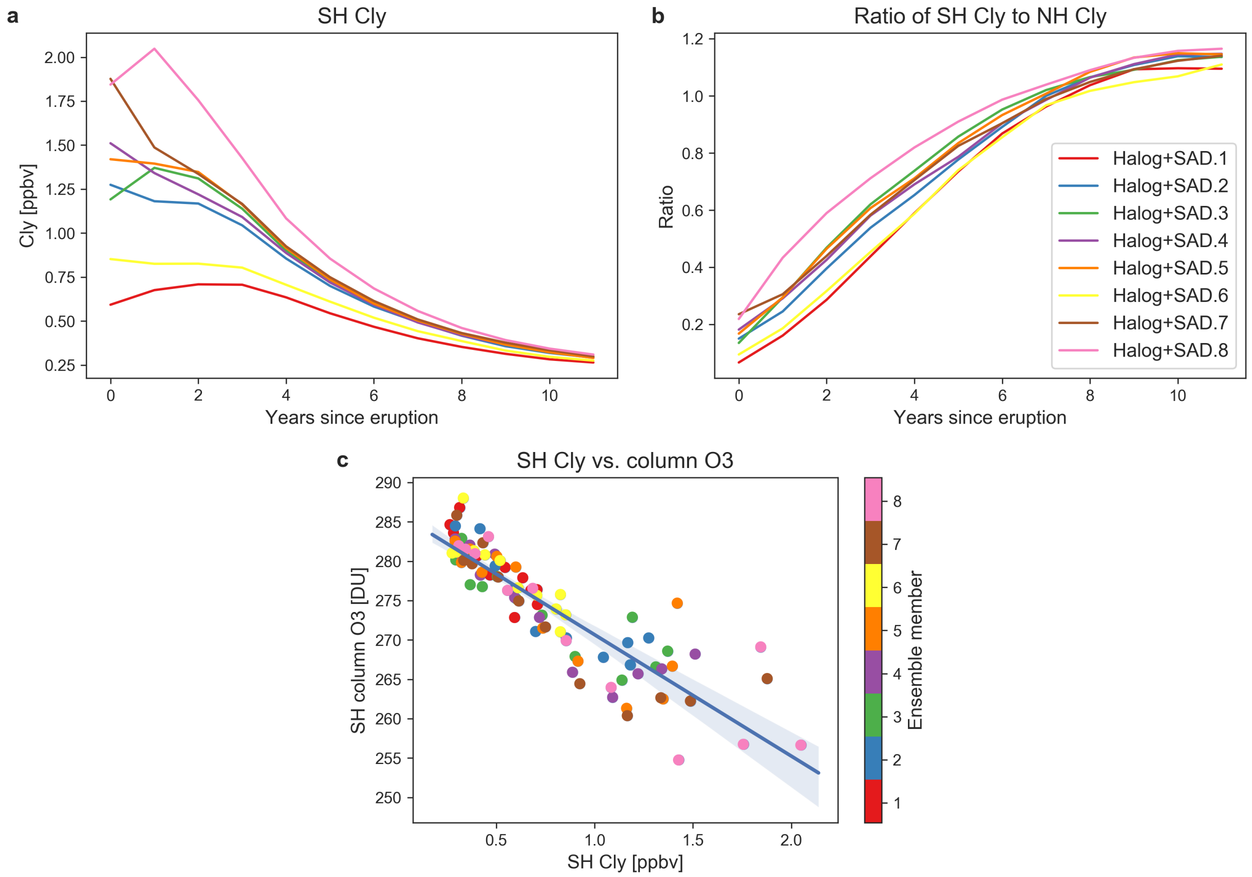

Figure S3: Annual mean average stratospheric concentration of total inorganic chlorine (Cl_y) for: (a) the Southern Hemisphere (SH). (b) Ratio of SH to NH Cl_y concentration. (c) Scatter plot of annual mean SH Cl_y vs. annual mean SH column ozone.

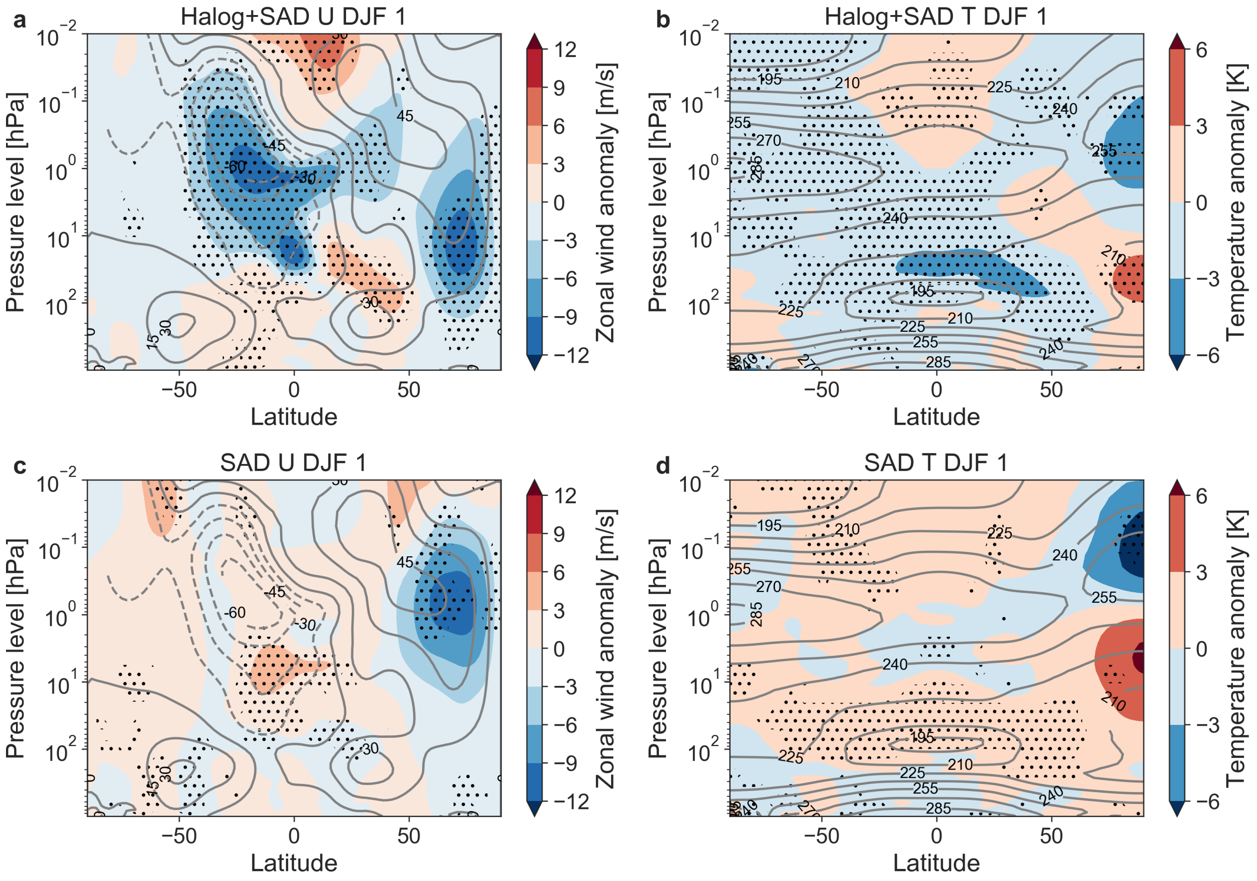

Figure S4: Ensemble mean zonal wind (a,c) and temperature (b,d) response during the first post-eruption DJF in the Halog+SAD (a,b) and SAD (c,d) forcing experiments. Gray contours represent the control climatology with intervals of 15 m/s and 15 K respectively. Stipling indicates where changes are significant to the 95% level.

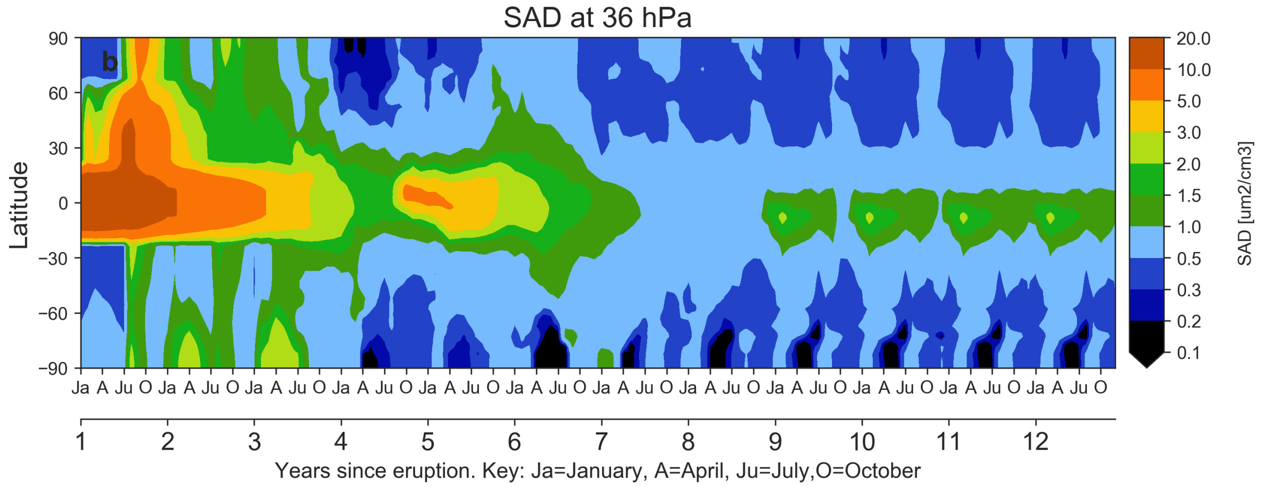

Figure S5: Aerosol surface area density (SAD) used as volcanic forcing in the model experiments, shown at 36 hPa. The SAD data from the April 1982 El Chichon eruption [SPARC, 2006], were shifted three earlier for a January eruption.

References

[SPARC, 2006] SPARC (2006). SPARC Assessment of Stratospheric Aerosol Properties (ASAP). Technical report, SPARC.
